# Molecular diagnosis of strongyloidiasis in tropical areas: a comparison of conventional and real-time polymerase chain reaction with parasitological methods

**DOI:** 10.1590/0074-02760140371

**Published:** 2015-04

**Authors:** Fabiana Martins de Paula, Fernanda de Mello Malta, Priscilla Duarte Marques, Renata Barnabé Sitta, João Renato Rebello Pinho, Ronaldo César Borges Gryschek, Pedro Paulo Chieffi

**Affiliations:** 1 Instituto de Medicina Tropical; 2 Departamento de Moléstias Infecciosas e Parasitárias; 3 Departamento de Gastroenterologia, Hospital das Clínicas, Faculdade de Medicina, Universidade de São Paulo, São Paulo, SP, Brasil; 4 Faculdade de Ciências Médicas da Santa Casa de São Paulo, São Paulo, SP, Brasil

**Keywords:** Strongyloides stercoralis, molecular diagnosis, conventional PCR, real-time PCR

## Abstract

This study aimed to evaluate the use of conventional polymerase chain reaction (cPCR) and real-time quantitative PCR (qPCR) in the diagnosis of human strongyloidiasis from stool samples in tropical areas. Stool samples were collected from individuals and were determined to be positive for Strongyloides stercoralis (group I), negative for S. stercoralis (group II) and positive for other enteroparasite species (group III). DNA specific to S. stercoralis was found in 76.7% of group I samples by cPCR and in 90% of group I samples by qPCR. The results show that molecular methods can be used as alternative tools for detecting S. stercoralis in human stool samples in tropical areas.

Strongyloidiasis is an intestinal infection that is caused by the nematode *Strongyloides stercoralis *and is estimated to affect 30-100 million people worldwide (Olsen et al. 2009). In endemic areas, chronic infections can be maintained asymptomatically for decades through cycles of autoinfection. Furthermore, this helminthiasis presents the potential for hyperinfection and disseminated disease in immunocompromised patients (Mejia & Nutman 2012). Therefore, the development of highly sensitive diagnostic tests to detect chronic cases of strongyloidiasis is crucial to prevent life-threatening infections (Paula & Costa-Cruz 2011).

DNA detection is increasingly being used to identify parasites and it could solve current problems in the diagnosis of human strongyloidiasis (ten Hove et al. 2009). However, the advantages and disadvantages of conventional polymerase chain reaction (cPCR) and real-time quantitative PCR (qPCR) in the diagnosis of human strongyloidiasis have not been discussed in the literature. Thus, this study aimed to evaluate cPCR and qPCR regarding the detection of *Strongyloides stercoralis* DNA from stool samples in tropical areas.

Stool samples from 100 patients (45 males and 55 females, average age 45.6 years) seen at the Clinics Hospital of Medical School of University of São Paulo (HC-FMUSP) were collected and immediately examined using the methods of Lutz (1919) and Rugai et al. (1954) and by agar plate culture (Koga et al. 1991). The samples were divided into three groups: group I (n = 30), stool samples positive for *S. stercoralis* by at least one parasitological method; group II (n = 40), stool samples negative by all parasitological methods, and group III (n = 30), stool samples positive for other enteroparasites. The samples in group III were positive for *Schistosoma mansoni* (n = 7), hookworm (n = 4), *Ascaris lumbricoides* (n = 3), *Hymenolepis nana* (n = 1), *Enterobius vermicularis* (n = 1), *Giardia lamblia* (n = 4), *Endolimax nana* (n = 4), *Blastocystis* sp. (n = 2), *Entamoeba coli* (n = 1) and the species found in three poly-infected samples (*S. mansoni, A. lumbricoides, E. coli, Blastocystis* sp. and *E. nana*; hookworm, *S. mansoni, E. nana *and* Blastocystis *sp.; hookworm, *E. coli* and *Entamoeba histolytica*/*Entamoeba dispar*). This study was approved by the Research Ethical Committee of the HC-FMUSP (protocol 0123/10).

After the parasitological methods were performed, an aliquot of the stool sample was preserved in 70% ethanol and maintained at 4ºC until subsequent DNA extraction. Approximately 500 mg of stool was shaken vigorously and centrifuged. The resulting pellet was washed twice in phosphate-buffered saline (0.01 mol/L, pH 7.2), after which DNA extraction was performed using the QIAamp Stool Mini Kit (Qiagen, Germany). The extracted DNA was quantified using a NanoDrop ND-1000 UV-VIS spectrophotometer v.3.2.1 (NanoDrop Technologies, USA).

The primers Stro18S-1530F and Stro18S-1630R were used to amplify DNA from *S. stercoralis*, as described by Verweij et al. (2009). The cPCR reaction was performed in a final volume of 25 µL, using 10 ng of DNA. The PCR products were loaded on a 2% agarose gel, stained with ethidium bromide and separated by electrophoresis in 1X TAE buffer. DNA extracted from filariform larvae of *S. stercoralis *obtained from positive agar plates was used as a positive control. The human β-globin gene was used as an internal control (Saiki et al. 1985) and distilled water was used as a negative control.

qPCR was performed according to the protocol described by Verweij et al. (2009). For each reaction, 10 ng of DNA and the Stro18S-1586T probe (2.5 pmol/µL) were combined in a final volume of 12.5 µL. The TaqMan^(r)^ exogenous internal control kit (Applied Biosystems, Life Technologies, USA) was used as a control. The qPCR reactions were performed using an ABI 7300 Real Time PCR System (Applied Biosystems, Life Technologies).

Statistical analyses were performed using GraphPad (GraphPad Software, USA). The results of each method were compared with those of the parasitological techniques and the degree of agreement between the techniques was verified with the kappa (ҡ) coefficient and the respective 95% confidence interval.

Among stool samples in group I, 14 (46.7%) were positive for *S. stercoralis* by the Lutz method, 19 (63.3%) were positive by the Rugai method and 27 (90%) were positive by agar-plate culture. Studies have demonstrated the high sensitivity of agar-plate culture, even when only a few worms are present (Inês et al. 2011).

Specific amplification of *S. stercoralis* DNA was observed (Table). In group I, *S. stercoralis* DNA was amplified in 23 of 30 samples (sensitivity, 76.7%). In group II, DNA was detected in seven of 40 samples. In group III, DNA was amplified in samples positive for hookworm (n = 2) and in poly-infected samples (n = 2). Considering groups II and III together, the specificity of cPCR was 84.3%.

**TABLE:** Molecular methods for the detection of Strongyloides stercoralis

		cPCR n (%)^*a*^			qPCR n (%)^*b*^	
Samples	n	Positive	Negative		Positive	Negative
Group I	30	23 (76.7)	7 (23.3)		27 (90)	3 (10)
					(27.83-39.2)^*c*^	NA
Group II	40	7 (17.5)	33 (82.5)		7 (17.5)	33 (82.5)
					(33.13-38.39)^*c*^	(40.41-46.89)^*c*^
Group III	30	4 (16.7)	36 (83.3)		3 (10)	27 (90)
					(30.7-37.08)^*c*^	(39.64-44.73)^*c*^

a : kappa coefficient (ҡ) = 0.710 (0.565-0.855)

b : ҡ = 0.587 (0.417-0.757)

c : threshold cycle value range; cPCR: conventional polymerase chain reaction; NA: no amplification; qPCR: quantitative PCR.

The sensitivity of qPCR was determined with a serial dilution curve (10^-1^ to 10^-10)^, performed in triplicate, using DNA from the infective larvae of *S. stercoralis* at an initial concentration of 21.39 ng/µL. Samples with a cycle threshold (C*_T_*) value > 39.2 were considered negative to prevent false-positive results because the samples are from tropical areas.* S. stercoralis-*specific amplification using qPCR was observed in 27 of 30 samples from group I. In group II, DNA amplification was observed in seven of 40 samples. In stool samples from group III, DNA amplification was observed in several samples, including samples positive for hookworm (n = 2) and in poly-infected samples (n = 1). The sensitivity and specificity of qPCR were 90% and 85.7%, respectively.

The intermittent nature of the elimination of larvae and the occasional presence of dead larvae may explain the false-negative parasitological results in groups II and III and may also explain the positive results found using molecular techniques. As reported previously, the analysis of consecutive samples can increase the sensitivity of parasitological methods (Khieu et al. 2013). Furthermore, intestinal parasites are widespread in humans living in tropical and subtropical countries, which present favourable climatic conditions for their development. In Brazil, epidemiological studies indicate that significant portions of the populations analysed were positive for parasites (Gonçalves et al. 2011). DNA competition in samples from endemic areas frequently leads to non-specific amplification.

The results obtained using molecular methods in this study identified false-negative results in some specimens. A possible explanation for this could be that amount of faecal sample is greater are analysed using parasitological techniques (Verweij et al. 2009). In a recent study, Moghaddassani et al. (2011) reported 100% sensitivity for cPCR compared with agar-plate culture; however, these authors used 3 g of each faecal sample for DNA extraction, whereas 500 mg of each faecal sample was used in this work.

In the present study, a comparison of the results showed that qPCR was more sensitive and specific than cPCR, presenting better ҡ values (0.710), considering the parasitological methods to be the gold standard. Disagreements in the results were observed in all groups; some samples were negative by cPCR, but were positive by qPCR and the opposite was observed, as well. Different results for qPCR were reported by Verweij et al. (2009) and Schär et al. (2013), with lower sensitivity and higher specificity.

Real-time qPCR entails higher overall costs, which make cPCR a more cost-effective methodology. If a laboratory has good infrastructure and the reagents required for qPCR, then this technique becomes very attractive. However, in countries where resources are limited, cPCR can be used as an alternative for the detection of *S. stercoralis*. The development and implementation of new detection methods involving molecular techniques are of fundamental importance for the accurate detection of parasites by PCR in the future. Molecular techniques have increasingly been used as diagnostic tools for parasites; however, new studies are of fundamental importance for discovering new target sequences and also for improving the interpretation of results, especially for determining the C*_T_* values to be used as cut-off values.

This study demonstrated that molecular methods are sensitive and specific for detecting *S. stercoralis *in human stool samples. Thus, cPCR, as well as qPCR, can be used as alternative tools for detecting *S. stercoralis* in human stool samples in tropical areas.
